# Transparent reporting in forensic Science: Exploring its meaning and challenges

**DOI:** 10.1016/j.fsisyn.2025.100630

**Published:** 2025-07-18

**Authors:** Kristy A. Martire

**Affiliations:** School of Psychology, The University of New South Wales Sydney, NSW, 2052, Australia

## Abstract

The forensic and scientific communities widely endorse transparency as a core principle and fundamental obligation of forensic science reporting. Yet the definition of transparency - ironically – remains opaque. This ambiguity impacts scientist's ability to fulfill their obligations when reporting forensic findings to justice systems as the primary consumer. Applying Elliott's 2022 taxonomy of transparency clarifies the issue, revealing that transparency is central to achieving Reliability, Assessment, Justice, Accountability and Innovation goals. It involves disclosing information about the scientists' Authority, Compliance, Basis, Justification, Validity, Disagreements, and Context, and shows that the audiences for these disclosures includes not only primary consumers, but also a range of agents, actors, and stakeholders. This complexity creates a multidimensional challenge for scientists and forensic science service providers, requiring a careful balance between competing demands. Templates can mitigate some of these challenges, but must be coupled with ongoing collaboration among forensic scientists, legal stakeholders, and institutional bodies to ensure that reporting practices evolve in line with professional obligations, scientific rigor and the realities of forensic practice.

Forensic and scientific communities widely endorse transparency as a core principle and fundamental obligation when reporting the results of forensic examinations. Diverse forensic science organisations, legal institutions and scholars explicitly call for transparency from forensic scientists [[1], [2], [3], [4], [5], [6], [7], [8], [9]], while others support it implicitly through disclosure requirements and expectations about clear, open and comprehensive reporting practices [[10], [11], [12], [13]]. This broad support illustrates the fundamental need for transparency in forensic science reports. Yet, it is not merely one reporting obligation among many. Unlike other obligations, transparency also serves as an organising principle - that shapes report content, purpose and form on one hand, while generating complexity on the other: “*scientists can be transparent about many different things, for many different reasons, on behalf of many different stakeholders*” [14]. As such, delivering transparency poses a unique and complex challenge.

Among forensic scientists, transparency commonly equates to an openness about the basis for their conclusions, as well as the reliability and limitations of those conclusions given the underlying methods and procedures. Even so, closer examination of the concept reveals both diversity and nuance – showing that transparency serves an array of goals for a broad spectrum of audiences. This definitional ambiguity motivates the present paper. A lack of clarity about what transparency entails – its purpose, content, mechanisms, costs, and intended audience – limits forensic scientists' ability to deliver transparency and meet their reporting obligations. Moreover, in the absence of a clear understanding of transparency as a guiding principle, efforts to define, advocate for, debate and deliver ‘good’, ‘effective’ or ‘adequate’ reports risk being arbitrary and disconnected from established norms and obligations.

This paper examines how transparency is described and discussed to clarify expectations about the content of forensic science reports. It moves beyond the basic idea that forensic scientists should report transparently, to tease apart the concept of transparency itself, revealing its meaning for forensic reporting. Doing so opens new avenues for improving written reports as the primary means of communication between forensic scientists and their diverse audiences [15,16] - thereby helping forensic scientists to craft reports that meet professional obligations and communicate clearly and efficiently.

## 1 A taxonomy of transparency

Recognising the complex and often ambiguous nature of transparency in mainstream science led philosopher Kevin Elliott to develop a framework for systematically exploring the Why, What, Who and How of transparency, as well as the associated risks [14]. The remainder of this paper uses Elliott's taxonomy to expose the complexity of the construct for forensic science reporting, along with the challenges practitioners and service providers face in meeting transparency obligations.

### 1.1 Why write transparent reports?

To deliver reports that meet transparency obligations and expectations, forensic scientists must first understand the rationale behind the requirement: What are forensic science reports meant to achieve? [14] Without clarity about this purpose - or the criteria for success - efforts to decide what should be disclosed, to whom, and in what way risk being unfocused or ineffective.

The literature on transparency in forensic science provides scientists with a range of definitions to guide their report writing. Some of these purposes align with mainstream scientific norms. However, others reflect the specific features of forensic science practice and its unique legal context. Broadly, the literature identifies five core purposes of transparency: 1) Reliability, 2) Assessment, 3) Justice, 4) Accountability, and 5) Innovation.

#### 1.1.1 Reliability

The first of the core purposes relates to the accuracy and dependability of scientific conclusions. While many scientists are intrinsically motivated to uphold high standards, the knowledge that their methods, results and reasoning will be exposed to external scrutiny can further motivate practitioners to employ the most defensible methods, rigorously check their work, and ensure their findings are valid and robust [17,18]. In this way, transparency reinforces good scientific practice. It also informs judgments about trustworthiness, competence and the strength of conclusions by making visible potential conflicts of interest or sources of bias [3,5,[18], [19], [20], [21]]. As Carr and colleagues suggest, “*trust cannot be assumed, it requires the provision of intelligible information to demonstrate that a person is trustworthy*” [18].

#### 1.1.2 Assessment

A second purpose frames transparency as a mechanism enabling external parties to evaluate and apply forensic findings. As noted in *Davie v Lord Provost* “*[T]he bare ipse dixit of a scientist, however eminent, upon the issue in controversy, will normally carry little weight, for it cannot be tested by cross-examination nor independently appraised …”* [22]. By prompting disclosures about the actions performed by scientists, the findings obtained, their basis and limitations, transparency facilitates an unbiased and independent assessment of forensic scientists’ work [3,4,6,18,19,[23], [24], [25], [26]]. These disclosures enable consumers – such as investigators, legal professionals, and courts - to assess the strength of the evidence and its weight in resolving legal matters [3,5,[26], [27], [28], [29], [30]]. Without a complete and thorough record of procedures and outcomes [3,13,24], audiences are at risk of misunderstanding findings or ineffectively testing them - undermining the accuracy and fairness of justice processes [3,6].

#### 1.1.3 Justice

A third perspective sees transparency as essential for the equitable and legitimate administration of justice. Many legal systems recognise the fundamental right of defendants to examine and challenge the evidence presented against them [[31], [32], [33], [34], [35]]. Disclosures not only serve this principle, but also foster compliance with legal procedures, rules of evidence, and admissibility determinations [3,5,6,24,30,[36], [37], [38]]. A failure to disclose relevant information risks undermining these legal protections. Indeed, “*most jurisdictions require that expert testimony be based on sufficient facts or data, generally accepted methods, and reliable application of facts to methods*” [6]. By revealing such information, transparency helps prevent sub-standard, inappropriate, or inequitable practices from taking root, and protects public confidence in both forensic science services and justice systems as a whole [18,24,38,39].

#### 1.1.4 Accountability

Transparent reporting serves a fourth purpose by holding forensic scientists accountable for their professional conduct and the quality of their work [19,24]. By clearly stating their values, scientists and service providers enable external parties to judge whether those values align with the broader expectations of institutions, communities and justice systems they serve [18,25]. Institutional arrangements – including standard operating procedures, procurement protocols, resource limitations, workloads, and workplace culture - shape how forensic scientists approach, deliver and interpret their work. Transparency exposes these influences, making it possible to evaluate how they affect outputs. Any misalignment between the values of end-users and the realities of forensic science practice risk undermining the credibility and value of forensic science evidence [17,18,38].

#### 1.1.5 Innovation

A fifth perspective characterises transparency as a way to propel scientific rigor and innovation. Openness is a hallmark of scientific endeavour, an integral component of good scientific practice, and the foundation of beneficial interactions between science and society [25,[39], [40], [41], [42]]. By providing detailed information about analytical methods, data and results, transparency facilitates progress through validation, replication or refinement of findings, as well as the development of cutting-edge technologies and techniques [13,14,19,25,38,43,44]. Without transparency, forensic science risks stagnation, limiting responses to emerging policing needs, and preventing the fulfilment of responsibilities surrounding community safety and the pursuit of justice.

### 1.2 What information should transparent reports contain?

Once identified, a reports’ purpose should guide clear, principled content decisions [14]. Given the volume and complexity of information forensic scientists hold, not everything they know or consider during an analysis can realistically be included in every report. The challenge lies in identifying what information is necessary to serve the intended purpose. Failure to do so risks producing reports that are either inadequate or dense, complex, and ineffective.

The literature points to seven kinds of information forensic scientists are expected to disclose: 1) Authority, 2) Compliance, 3) Basis, 4) Justification, 5) Validity, 6) Disagreements, and 7) Context. As with the purposes described above, some of these core content areas reflect the norms and expectations of mainstream science, while others originate from the unique forensic operational environment, such as criminal procedure rules, practice directions, and discipline-specific guidance documents [1,13,26,36,45,46].

#### 1.2.1 Authority

Details about expertise and credibility help determine whether a scientist has the authority to contribute meaningfully to forensic processes. Information about qualifications, accreditation, training, study, experience, proficiency, and competence all contribute to this assessment [3,7,47]. Similarly, outlining the nature and scope of expertise clarifies whether the scientist stays within the bounds of their professional knowledge when offering their opinions [3,5,18,25,38,45,46]. This category of disclosure also includes information about relationships or potential conflicts of interest that may affect the objectivity and fairness of their practices or conclusions [19,24]. These details support Reliability, Assessment and Justice goals, and are especially important for expert admissibility determinations.

#### 1.2.2 Compliance

Regulatory agencies and legal institutions commonly demand that forensic scientists provide information about compliance with relevant rules and procedures [3,5,8,18,24,45,46]. Formal statements of compliance with codes, accreditation requirements, certification obligations, national and international standards of practice, and regulations all serve a number of purposes, but they align best with Accountability and Justice goals.

#### 1.2.3 Basis

Both legal and scientific communities expect that scientists will clearly explain the basis of their opinions. This includes information about the materials, methods, techniques, procedures, data and results, as well as how these align with best practice or standard operating procedures [3,[5], [6], [7], [8], [9],^17^,[23], [24], [25],^38^,^41^,[45], [46], [47]]. In addition, forensic scientists should describe the completeness and quality of the evidence underpinning their judgements [24,46], and clarify any influence of external facts, opinions, assumptions or background knowledge [7,8]. This category of disclosures also includes the results of review or verification procedures [9,25,46,48]. Together, these elements speak to the Reliability of the opinion, enable an Assessment of evidence value, and support Innovation.

#### 1.2.4 Justification

The literature flags reasoning and interpretations as another key area of disclosure. This includes the specification of all opinions and conclusions [1,5,13,23,24], as well a detailed explanation of the reasoning and interpretations leading to each conclusion - for example about the questions under investigation and the rationale driving key decisions. This may involve the selection of analytic approach, the structuring of information to support analysis, the methods for formulating, verifying, and weighting conclusions, and the influence of the scientists training, study and experience on both approach and outcome [1,5,9,17,18,23,25]. In addition to serving Reliability, Justice, Accountability and Innovation goals, these disclosures are vital to critical Assessments of evidence value.

#### 1.2.5 Validity

Calls for forensic scientists to disclose the validity and limitations of their methods and conclusions feature prominently in the literature. This topic covers details about the legitimacy of methods as applied [26], as well as caveats, conditions or qualifications that impact conclusions, materials or methods in an instant case, and the confidence or precision of the findings [3,5,13,17,18,24,27,[45], [46], [47]]. The Presidents’ Council of Advisors on Science and Technology (PCAST) and others also insist that scientists provide evidence concerning the foundational validity of their work [5,18,24,26,47]. This may involve sharing information about known limits or uncertainties generally associated with procedures, methods, generalisations, and inferences [3,5,8,17,24,27,30,46,49] in addition to principles, knowledge, theories and research. For example, describing the rareness or commonness of particular traces, the identification paradigm, and citations to the relevant literature generally underpinning the scientists work [5,18]. Information about validity and its limits not only furthers Justice and Assessment goals, but also supports Reliability and Innovation.

#### 1.2.6 Disagreements

Numerous sources identified disagreements as a critical area of disclosure. This may include disagreements between scientists in a specific case, or they may relate to different perspectives or approaches adopted more broadly in the field. For instance, verification or independent peer review processes may reveal discrepancies that scientists should disclose [7,9,48]. Where disagreements arise as a result of variations in the methods, procedures, or analysis, scientists are also asked to explain the basis and significance of those disagreements [5,18,23,24,46]. For example, by comparing the scientists’ approach to generally accepted best practices and by highlighting the costs and or benefits of any departures. This information primarily supports Assessment and Justice, while also contributing to Accountability and Innovation.

#### 1.2.7 Context

Commentators increasingly encourage forensic scientists to disclose the contextual factors surrounding analysis and interpretation. Details that clarify what was known, by whom, and at what stage of the process are critical, not only for assessing whether findings may have been based on incorrect information, but also for identifying the influence of task-irrelevant contextual information or the risk of contextual bias [5,7,9,20,21,50]. The National Commission on Forensic Science distinguishes between task-relevant and task-irrelevant information and recommends that conclusions be based only on the former [51]. Scientists are also called on to describe institutional factors shaping their professional practice. For example, relating to standard operating procedures, corporate values and priorities, procurement rules and restrictions, workplace culture, contributions to the work product from other providers, and general or specific information about quality systems and arising issues [18,30]. This information serves all five transparency goals.

### 1.3 Who is the audience for transparent reports?

The next key insight from Elliott's framework concerns the central role of the intended audience in shaping transparency. Identifying the relevant audience helps scientists determine what kinds of disclosures are necessary, and why [14]. Passalaqua and colleagues [19] classify the audiences for forensic science reports into three main groups: the justice system as the *primary consumer*; victims, families and communities as *agents* who benefit from forensic services; and investigators, lawyers, judges, juries and defendants/litigants as *actors* within the justice system. In addition, Georgiou and colleagues [27] identify those with an interest in the product as *stakeholders*, who we can further divide into *Individual stakeholders* - such as the scientists performing the analysis, academics and other scientists - and *institutional stakeholders,* including regulators, service providers and government bodies invested in forensic scientific practice.

Importantly, these diverse groups value different transparency goals. Judges, defendants and other legal actors primarily focus on admissibility and fairness (Justice). Institutional stakeholders such as regulators and service providers prioritise compliance and oversight (Accountability). Individual stakeholders – like academics and other scientists – emphasize methodological transparency and replicability (Reliability & Assessment). Whether a report delivers transparency therefore depends on its audience and purpose. Only by carefully examining transparency do we see the importance of audience-centred reporting and the value of tailoring disclosures to stakeholder needs.

### 1.4 How can reports deliver transparency?

After identifying the intended audience for transparent forensic reports, the purposes they will serve, and the information they should contain, the next consideration is how to communicate that information effectively [14]. Successful communication relies on careful consideration of reporting parameters [23], including timeframes, messengers, mechanisms and venues.

Beginning with timeframes, disclosures must occur when they are most useful to the intended recipients and goals. Prior to analysis scientists may need to share the formulation for their analytic plan or the reasoning underpinning their hypotheses to ensure their findings are relevant. During analysis preliminary results and associated caveats or contingencies will shape investigations. After the analysis, formal statements of compliance, data, results, opinions and disagreements determine the strength of prosecutions; and after the case is concluded, aggregated information about quality assurance incidents or validation results inform regulation and provide long-run feedback about evidence reliability. As with other aspects of disclosure, choices about timing will also be affected by other considerations. For example, annual disclosures are well suited to Accountability gaols, whereas as Assessment and Justice goals are better served by reporting timelines that coincide with the analytic process.

The choice of messenger is another important part of meeting transparency obligations. In most cases, the scientist completing the work is the primary messenger [23], providing details of their analysis to end-users. However, the responsibility to disclose extends to other messengers. For example, although they do not necessarily prepare court-going reports, quality assurance managers and service providers carry primary responsibility for making the disclosures about systems, compliance and oversight that serve Accountability goals [30]. Selecting the appropriate messenger therefore depends on the nature of the information, its purpose, and audience needs.

The mechanisms of disclosure also require careful consideration. Messengers can use various mechanisms to achieve transparency including via peer reviewed journal articles, quality assurance registers, and publicly available policy documents. However, this paper focuses specifically on written reports as the primary mechanism of disclosure due to their central role. While this emphasis narrows the scope of our discussion to a single disclosure format, significant variation exists even within forensic science reports, which commonly take various forms and include multiple components [52].

For example, investigatory or intelligence reports - which shape the early stages of police work – differ from finalised “court-going” reports - which inform prosecution, defense, and fact-finding by judges and juries. Even among finalised reports, the level of detail varies substantially. Sometimes scientists use brief certificates or intentionally streamlined statements to convey their findings [24]. In other cases, scientists provide end-users with comprehensive reports including detailed annexures, or will supplement their reports with exhaustive case notes upon request [5,23,24,40,47]. Crucially, determining the appropriateness of a particular reporting format or level of detail depends on the intended purpose and audience. If the report does not suit its pre-determined goal then that report is not fit-for-purpose and a different form or level of reporting is needed [24].

Finally, forensic scientists must also consider where they will share their knowledge. Adversarial or inquisitorial legal proceedings are usually the primary venue of disclosure for most forensic scientists. However, scientists’ reports may contribute to quality assurance processes, inform regulatory decisions, support academic research, address private queries, or assist commercial investigations. These venues generate different expectations about the audience, timing, purpose and content of disclosures. The methods that successfully deliver transparency in one context, may fall short in another. Forensic scientists must tailor the timing, detail, and format of disclosures to the specific goals and expectations of each context to meet transparency obligations effectively.

### 1.5 Collateral costs & benefits

Of course, efforts to provide transparency may also have collateral consequences [14]. These unintended effects fall into four broad categories: cognitive impact, resource allocation, operational efficiency, and informational flow. First, poorly designed reports may have unintended impacts on cognition. Rather than enhancing clarity, detailed disclosures may cause confusion and misunderstanding [23,25,30], overburdening, inundating or overwhelming audiences, particularly those without technical expertise [3,24]. Such outcomes clearly undermine transparency goals by complicating evidence assessments and potentially compromising the fair administration of justice.

Second, transparency will almost inevitably increase resource demands. Producing and reviewing more comprehensive reports is both time consuming [5] and costly [25], and could slow or even obstruct investigation and analysis - particularly in under-resourced laboratories. In systems already stretched thin, transparency might therefore conflict with broader objectives, such as timely service to communities and justice. However, there are also collateral benefits to offset these costs. Transparency offers a mechanism for streamlining forensic science practice through more efficient reviews, and minimising unnecessary duplication or reanalysis. It also fosters collaboration [25,38] by building a common understanding of the current state of the science.

Third, transparency can reduce operational efficiency by increasing scrutiny of, and accountability for, forensic scientists and the upstream processes they rely on [25,41]. While it is clear that justice actors are entitled to examine every aspect of the evidence in a case – and should be provided the information necessary to do so - this heightened scrutiny can contribute to occupational stress, lower morale, increase attrition, and potentially weaken the profession [5,25,53]. These are serious risks. Yet transparency also helps to address them. As scrutiny becomes normalised, professional expectations and practices will evolve – building a workforce more prepared for, and more resilient to, these unique accountability obligations.

Fourth, the provision of additional information - particularly regarding the weaknesses and limitations of methods or analyses - may invite unwarranted scepticism among consumers [47]. To be clear, transparency about such limitations is essential and should not be avoided. In many cases, these types of disclosures should justifiably prompt a re-evaluation of the evidence, and may lead to reasonable restrictions on certain evidence or expert testimony [3]. However, disclosures may also be misinterpreted, strategically misused [30,53], or result in disproportionate reactions. For example, detailed explanations of methodological limitations or error rates may fuel inaccurate perceptions of unreliability even when findings are robust and well-supported. This kind of overcorrection risks distorting the evidentiary value of forensic reports, undermining confidence, reducing legal weight, and potentially contributing to unjust outcomes. It may also stifle innovation and slow the uptake of emerging technologies that could otherwise enhance the field. These risks are difficult to eliminate entirely, particularly in adversarial legal setting where strategic misrepresentation is always possible – but they are not grounds for withholding required information.

In fact, transparency also offers benefits in this context. By clearly disclosing limitations, forensic scientists help set realistic expectations for evidence interpretation among legal consumers, guard against overstated confidence, and empower decision-makers to weigh findings appropriately. In this way, transparency can shift expectations towards more realistic modesty – better aligning forensic and mainstream scientific norms, and improving justice outcomes, even if there is some misuse or overcorrection along the way. There is even evidence that transparent practices enhance credibility [38,54], and protect scientists by facilitating pre-emptive disclosures of details to mitigate later legal challenges [3]. Transparency also supports the development of actionable forensic intelligence that takes appropriate account of the risks and limitations of forensic findings, ensuring judicious applications [30].

Finally, disclosures create risks to information flow, including threats to security, privacy, and intellectual property [6,25]. Unchecked, these issues could stifle innovation by discouraging investment in technologies that require early disclosure, reducing willingness to share advancements, and creating uncertainty that hinders long-term research and development efforts. Yet, such challenges are not unique to forensic science. Many fields face intellectual property and confidentiality risks, which can be addressed through careful consideration of what needs to be disclosed, to whom and when, in order to protect against misuse.

Overall, when weighing these effects, it is important to note that Elliott's taxonomy places more emphasis on collateral costs than benefits at this point of the framework, because all potential downsides count as costs, but achieving the primary goals does not count as a *collateral* benefit. Nevertheless, transparency clearly does carry potential costs – some offset by its benefits, others requiring mitigation – and the obligation to be transparent remains.

## 2 Discussion

Although transparency is a regulated obligation for forensic scientists in many jurisdictions, the concept itself is ambiguously defined. Such ambiguity impacts scientist's ability to fulfil their obligations and produce reports that are fit-for-purpose. Elliott's taxonomy [14] helps us to understand what transparency entails for forensic scientists, clarifying the why, what, who and how, as well as the collateral costs and benefits associated with achieving it. Ultimately, doing so reveals the complex practical challenge confronting forensic scientists and forensic service providers.

Specifically, transparent reports can serve five different purposes, some of which are unique to the forensic environment, creating novel challenges. For instance, although transparency is vital for the critical assessment of both mainstream and forensic science, only forensic scientists are expected to write reports that will facilitate assessments of probative value. Similarly, while Accountability is important for both groups, forensic scientists are subject to a range of regulatory and ethical requirements, like expert codes of conduct and rules of evidence, that do not apply to mainstream scientists. Thus, this review demonstrates that the implementation of transparent reporting in the forensic sciences requires discipline-specific strategies and support. This could involve the development of disclosure policies, reporting platforms, guidance documents or stakeholder feedback to translate transparency principles into useable reporting practices.

Transparent reporting also calls on scientists to cover a vast array of content in a manner suitable for diverse audiences. Not only does this create a risk that scientists and service providers will become overwhelmed by the practical realities of transparent reporting, the differences between audience groups are not easily addressed – they reflect divergent skills and views about the fundamental form and function of transparency which need to be accommodated. For instance, victims, defendants and judges need transparency so they can test the evidence and apply the law; scholars and other scientists value transparency as way to understand and strengthen the science; while institutional stakeholders require transparency so they can protect the integrity of forensic services. Facilitating all of these outcomes involves a range of delivery timelines, methods, and messengers.

Yet despite this convoluted web of interrelated needs, Elliott's [14] taxonomy also provides the clarity needed to guide transparency initiatives. In particular, the proceeding review highlights the central role of justice systems as the primary consumers of forensic science reports [19]. Since forensic findings only matter when they are admissible and probative [55], and forensic reports are already shaped by formal legal and procedural requirements [1,13,26,36,45,46], prioritising the Justice and Assessment goals [23] valued by justice systems serves as a powerful and efficient route to transparency. In fact, a thorough assessment of probative value requires disclosures in all seven key topic areas. Consequently, by focusing on the Assessment goal, scientists can simultaneously advance Justice, Reliability, Accountability, and Innovation aims. The trade-off is that producing such reports demands time and resources, and risks overloading scientists and end-users alike [3,5,[23], [24], [25],^30^,^41^,^53^]. Research shows that templates may be a viable means of mitigating many of these challenges.

Templates improve reporting quality across a range of fields [[56], [57], [58]] in part by ensuring reports consistently include the detailed descriptions to support evaluation. Templates also offer efficiencies by recycling uniform or slowly evolving content, such as standard operating procedures, methods, validity, error, and assumptions. Templates therefore ensure stable content is covered adequately, consistently and efficiently without requiring scientists to start afresh with each new report.

Templates offer further advantages by mitigating the cognitive burdens associated with transparency. By collaborating with psychologists, science communicators, linguists and educators, forensic scientists have the opportunity to design templates which minimise confusion and improve comprehension. These templates could incorporate features such as information hierarchies (e.g., headings and subheadings), navigation aids (e.g., tables of contents, glossaries), strategic emphasis techniques (e.g., the placement and order of key points), and a tiered reporting structure to help readers navigate efficiently through complex content arranged in a graduated manner – thereby allowing each audience to access the information at a level of detail appropriate for their needs.

Despite these benefits, we must also recognise that forensic service providers will require a suite of such templates to accommodate a variety of analyses and methods. Templates will also need to be flexible enough to accommodate case-specific details on an *ad-hoc* basis, such as the chain of custody, tests results, and basis of the opinion [19,23]. Thus, while the development of reporting templates poses logistical and resource challenges, they also offer a promising strategy to improve the consistency, clarity and feasibility of producing comprehensive forensic science reports.

Beyond these practicalities, Elliott's taxonomy also offers valuable insight into current debates about the appropriate scope and form of transparent reporting. A recent exchange in the *Journal of Forensic Sciences* between Thompson [59,60] and Kalafut, Curran, Coble and Buckleton [61], highlights this tension. One key issue was whether forensic DNA reports should present likelihood ratios (LRs) based solely on the analyst's best judgment about the number of contributors (NOC), or also include LRs calculated across a plausible range of NOC assumptions. Kalafut and colleagues supported a single LR reflecting the examiner's preferred NOC, citing confidence in the analysts' judgment and prioritising clarity and simplicity. Thompson, by contrast, argued that transparency requires disclosing the full range of defensible assumptions to reveal how such choices influence the result.

Elliott's taxonomy helps clarify what's at stake. While the narrower approach may reflect trust in the analyst and streamline reporting, it risks omitting important Basis, Validity, and Justification disclosures. The broader approach better supports Reliability, Assessment and Justice goals by equipping prosecutors and fact-finders – who must build and test cases – to evaluate the strength of the evidence independently, rather than deferring to expert opinion. This debate therefore highlights a potentially serious mismatch between the values of the audience and the reporting approach preferred by scientists.

The 2022 Australian Commission of Inquiry into Forensic DNA Testing in Queensland [62] presents a second example. Here, the Commission investigated whether describing samples with DNA quantitation values below 0.0088 ng/μL as “insufficient for further processing” or “no DNA detected” misled stakeholders. Importantly, the primary audiences here were police, prosecutors and families who relied on these reports to investigate crimes and understand the status of the case. Although efficient, the Commission found that for non-technical audiences, this phrasing obscured the fact that some samples might still yield interpretable results. Elliott's taxonomy clarifies why the reporting approach fell short: omitting information about Basis and Validity undermined the report's capacity to serve Assessment and Justice goals for the intended audience. The mismatch between the simplified language and the audiences needs contributed to serious consequences in this case: an estimated 30,000 reports required revision [62,63], delaying investigations, creating distress, and damaging public confidence in forensic services.

Together, these case studies show how Elliott's taxonomy can clarify, navigate, and potentially help resolve tensions by grounding them in core principles and audience needs. However, given the complexity of the issues involved, coordinated efforts – rather than isolated initiatives – are essential to making transparent reporting a reality. The National Institute of Standards and Technology (NIST), the European Association of Forensic Science Providers (EAFS), the National Institute of Forensic Science (NIFS) and the UK Forensic Science Regulator are well placed to lead efforts to fund, develop, evaluate and disseminate tools and templates to guide transparent reports. Sustained collaboration between forensic scientists, legal stakeholders, and institutions is also essential to keep reporting practices aligned with legal requirements, scientific standards, and the realities of forensic work. With collective effort and focused planning, the forensic science community can meet its transparency obligations and more effectively support the justice system.

## Generative AI disclosure

During the preparation of this work the author used OpenAI ChatGPT4 to improve clarity. After using this tool, the author reviewed and edited the content and takes full responsibility for the content of the publication.

## Declaration of competing interest

The authors declare that they have no known competing financial interests or personal relationships that could have appeared to influence the work reported in this paper.

## References

[1] CrimPR] C.P.R. (2020). *SI 2020/759*, Parliament of the United Kingdom of Great Britain and Northern Ireland.

[2] Biology Specialist Advisory Group, A (2024).

[3] Edmond G. (2016). Model forensic science. Aust. J. Forensic Sci..

[4] Association of Forensic Science Providers [AFSP] (2009). Standards for the formulation of evaluative forensic science expert opinion. Sci. Justice.

[5] Ballantyne K.N. (2024). A transparent approach: openness in forensic science reporting. Forensic Sci. Int.: Synergy.

[6] Sims D., Dove C., Frederick R. (2023). Transparency in forensic exams. Nev. LJ.

[7] Interpretation, E.W.G.o.H.F.i.F.D. (2024).

[8] Examination, E.W.G.f.H.F.i.H. (2021).

[9] Analysis, E.W.G.o.H.F.i.L.P. (2012).

[10] (2014). The Australian New Zealand forensic science society inc. Code Prof. Pract. Members ANZFSS.

[11] Edmond G., Cunliffe E., Martire K., San Roque M. (2018). Forensic science evidence and the limits of cross-examination. Melb. Univ. Law Rev..

[12] Victoria S.C.o. (2017).

[13] National Research Council [NRC] (2009).

[14] Elliott K.C. (2022). A taxonomy of transparency in science. Can. J. Philos..

[15] Howes L.M. (2013). Forensic scientists' conclusions: how readable are they for non-scientist report-users?. Forensic Sci. Int..

[16] Rothwell T., White and third P. (2010). Crime Scene to Court: the Essentials of Forensic Science.

[17] Earwaker H., Nakhaeizadeh S., Smit N.M., Morgan R.M. (2020). A cultural change to enable improved decision-making in forensic science: a six phased approach. Sci. Justice.

[18] Carr S., Piasecki E., Gallop A. (2020). Demonstrating reliability through transparency: a scientific validity framework to assist scientists and lawyers in criminal proceedings. Forensic Sci. Int..

[19] Passalacqua N.V., Pilloud M.A., Belcher W.R. (2019). Scientific integrity in the forensic sciences: consumerism, conflicts of interest, and transparency. Sci. Justice.

[20] Dror I.E. (2018). Biases in forensic experts. Science.

[21] Cooper G.S., Meterko V. (2019). Cognitive bias research in forensic science: a systematic review. Forensic Sci. Int..

[22] Davie v Lord Provost, Magistrate and Councillors of the City of Edinburgh.

[23] Sjerps M.J., Berger C.E.H. (2012). How clear is transparent? Reporting expert reasoning in legal cases. Law Probab. Risk.

[24] Edmond G., Carr S., Piasecki E. (2018). Science friction: streamlined forensic reporting, reliability and justice. Oxf. J. Leg. Stud..

[25] Chin J.M., Ribeiro G., Rairden A. (2019). Open forensic science. J. Law Biosci..

[26] President's Council of Advisors on Science and Technology [PCAST] (2016). Forensic science in criminal courts: ensuring scientific validity of feature-comparison methods. Executive Office President.

[27] Georgiou N., Morgan R.M., French J.C. (2020). Conceptualising, evaluating and communicating uncertainty in forensic science: identifying commonly used tools through an interdisciplinary configurative review. Sci. Justice.

[28] Edmond G., Cunliffe E., Martire K.A., San Roque M. (2019). Forensic science evidence and the limits of cross-examination. Melb. Univ. Law Rev..

[29] Edwards H.H.T. (2019). Innocence Network Annual Conference.

[30] Heavey A.L., Turbett G.R., Houck M.M., Lewis S.W. (2022). Toward a common language for quality issues in forensic science. WIREs Forensic Sci..

[31] (1966). International Covenant on Civil and Political Rights (ICCPR).

[32] Davis R v (2008).

[33] Fourteenth Amendment to the United States Constitution. 1868, United States Congress.

[34] Sixth Amendment to the United States Constitution. 1791, United States Congress.

[35] Committee, U.N.H.R. (2007).

[36] Crown Prosecutors Service [CPS] (2010).

[37] Kirby M. (2009). Editorial-forensic evidence: instrument of truth of potential for miscarriage?. J. Law Inf. Sci..

[38] Chin J.M., Ibaviosa C.M. (2022). Beyond CSI: calibrating public beliefs about the reliability of forensic science through openness and transparency. Sci. Justice.

[39] Houck M.M. (2019). Open, transparent science helps promote justice. Forensic Sci. Int.: Synergy.

[40] Maskell P.D. (2023). A model of evaluative opinion to encourage greater transparency and justification of interpretation in postmortem forensic toxicology. J. Anal. Toxicol..

[41] Crispino F. (2022). Towards another paradigm for forensic science?. WIREs Forensic Sci..

[42] Jasanoff S. (2006). Transparency in public science: purposes, reasons, limits. Law Contemp. Probl..

[43] Vazire S., Holcombe A.O. (2022). Where are the self-correcting mechanisms in science?. Rev. Gen. Psychol..

[44] Andrew G. (2018). *Open Science by Design: Realizing a Vision for 21st Century Research* E. National Academies of Sciences, and Medicine (NASEM).

[45] Supreme Court of Victoria [Practice Note] (2014).

[46] [CrimPD], C.P.D. (2021).

[47] Summersby S. (2024). The effect of following best practice reporting recommendations on legal and community evaluations of forensic examiners reports. Forensic Sci. Int..

[48] Ballantyne K.N., Edmond G., Found B. (2017). Peer review in forensic science. Forensic Sci. Int..

[49] Sigman M.E., Williams M.R. (2023). Promoting transparency in forensic science by integrating categorical and evaluative reporting through decision theory. Frontiers in Anal. Sci..

[50] Curley L.J., Munro J., Dror I.E. (2022). Cognitive and human factors in legal layperson decision making: sources of bias in juror decision making. Med. Sci. Law.

[51] Science N.C.o.F. (2015).

[52] Howes L.M. (2015). A step towards increased understanding by non-scientists of expert reports: recommendations for readability. Aust. J. Forensic Sci..

[53] Busey T., Sudkamp L., Taylor M.K., White A. (2022). Stressors in forensic organizations: risks and solutions. Forensic Sci. Int.: Synergy.

[54] Fetterman A.K., Sassenberg K. (2015). The reputational consequences of failed replications and wrongness admission among scientists. PLoS One.

[55] Edmond G., Cole S., Cunliffe E., Roberts A. (2013). Admissibility compared: the reception of incriminating expert evidence (ie, forensic science) in four adversarial jurisdictions. U. Denv. Crim. L. Rev..

[56] Woolery L., Budish R.H., Bankston K. (2016).

[57] Dickerson E. (2017). Effect of template reporting of brain MRIs for multiple sclerosis on report thoroughness and neurologist-rated quality: results of a prospective quality improvement project. J. Am. Coll. Radiol..

[58] Alper D.P. (2020). Effect of a report template–enabled quality improvement initiative on use of preferred phrases for communicating normal findings in structured abdominal CT and MRI reports. Am. J. Roentgenol..

[59] Thompson W.C. (2023). Uncertainty in probabilistic genotyping of low template DNA: a case study comparing STRMix™ and TrueAllele™. J. Forensic Sci..

[60] Thompson W.C. (2024). Author's response. J. Forensic Sci..

[61] Kalafut T., Curran J.M., Coble M.D., Buckleton J., Thompson W.C., Commentary on (2023). Uncertainty in probabilistic genotyping of low template DNA: a case study comparing STRmix™ and TrueAllele™. J. Forensic Sci..

[62] Sofronoff W.K. (2022). Commission of Inquiry into Forensic DNA Testing in Queensland.

[63] Gillespie E. (2023).

